# Culture and genome-based analysis of four soil *Clostridium* isolates reveal their potential for antimicrobial production

**DOI:** 10.1186/s12864-021-08005-2

**Published:** 2021-09-22

**Authors:** Amila S. N. W. Pahalagedara, Ruy Jauregui, Paul Maclean, Eric Altermann, Steve Flint, Jon Palmer, Gale Brightwell, Tanushree Barua Gupta

**Affiliations:** 1 Food System Integrity team, Hopkirk Research Institute, AgResearch Ltd, Massey University, 4474 Palmerston North, New Zealand; 2 School of Food and Advanced Technology, Massey University, 4442 Palmerston North, New Zealand; 3 Data Science team, Grasslands Research Centre, AgResearch Ltd, Palmerston North, New Zealand; 4 Riddet Institute, Massey University, Palmerston North, New Zealand; 5 New Zealand Food Safety Science and Research Centre, Massey University, Palmerston North, New Zealand

**Keywords:** *Clostridium* spp., Antimicrobial, Biosynthesis gene clusters, Genome mining, Ribosomally synthesized post-translationally modified peptides, Non-ribosomal peptides

## Abstract

**Background:**

Soil bacteria are a major source of specialized metabolites including antimicrobial compounds. Yet, one of the most diverse genera of bacteria ubiquitously present in soil, *Clostridium*, has been largely overlooked in bioactive compound discovery. As *Clostridium* spp. thrive in extreme environments with their metabolic mechanisms adapted to the harsh conditions, they are likely to synthesize molecules with unknown structures, properties, and functions. Therefore, their potential to synthesize small molecules with biological activities should be of great interest in the search for novel antimicrobial compounds. The current study focused on investigating the antimicrobial potential of four soil *Clostridium* isolates, FS01, FS2.2 FS03, and FS04, using a genome-led approach, validated by culture-based methods.

**Results:**

Conditioned/spent media from all four *Clostridium* isolates showed varying levels of antimicrobial activity against indicator microorganism; all four isolates significantly inhibited the growth of *Pseudomonas aeruginosa*. FS01, FS2.2, and FS04 were active against *Bacillus mycoides* and FS03 reduced the growth of *Bacillus cereus*. Phylogenetic analysis together with DNA-DNA hybridization (dDDH), average nucleotide identity (ANI), and functional genome distribution (FGD) analyses confirmed that FS01, FS2.2, and FS04 belong to the species *Paraclostridium bifermentans*, *Clostridium cadaveris*, and *Clostridium senegalense* respectively, while FS03 may represent a novel species of the genus *Clostridium*. Bioinformatics analysis using antiSMASH 5.0 predicted the presence of eight biosynthetic gene clusters (BGCs) encoding for the synthesis of ribosomally synthesized post-translationally modified peptides (RiPPs) and non-ribosomal peptides (NRPs) in four genomes. All predicted BGCs showed no similarity with any known BGCs suggesting novelty of the molecules from those predicted gene clusters. In addition, the analysis of genomes for putative virulence factors revealed the presence of four putative *Clostridium* toxin related genes in FS01 and FS2.2 genomes. No genes associated with the main *Clostridium* toxins were identified in the FS03 and FS04 genomes.

**Conclusions:**

The presence of BGCs encoding for uncharacterized RiPPs and NRPSs in the genomes of antagonistic *Clostridium* spp. isolated from farm soil indicated their potential to produce novel secondary metabolites. This study serves as a basis for the identification and characterization of potent antimicrobials from these soil *Clostridium* spp. and expands the current knowledge base, encouraging future research into bioactive compound production in members of the genus *Clostridium*.

**Supplementary Information:**

The online version contains supplementary material available at 10.1186/s12864-021-08005-2.

## Background

Antimicrobials are widely used in medicine, food, and agriculture to kill or inhibit the growth of harmful microorganisms [1–3]. Antibiotics have saved lives of millions by controlling infectious diseases [3]. Food industry uses natural and synthetic antimicrobials to control food spoilage and pathogenic bacteria in various food products. However, there is a demand for novel natural antimicrobials for food preservation due to consumer concerns over the adverse health effects of synthetic antimicrobials [2]. The rapid emergence of antimicrobial resistant bacteria poses a challenge for currently available antimicrobial agents used for human and animal infections, and food preservation. The consumer demand and threat of bacterial resistance have emphasized the need for the identification of novel antimicrobial compounds, which may have applications in the food industry and/or medicine from natural sources such as bacteria [4, 5].

In the past few decades, bacteria have been considered to be the most promising source of antimicrobials [6]. Soil is one of the most complex ecosystems harbouring a large and diversified group of bacteria. Soil microorganisms have developed various strategies to survive and multiply under the variable abiotic and biotic conditions of soil. The production of bioactive molecules such as antimicrobials is a survival mechanism for these microorganisms [7]. Soil actinomycetes have been screened for antimicrobials and they produce more than 80 % of the known antimicrobials used in today’s medicine and other applications. However, soil anaerobes such as *Clostridium* species, which are metabolically diverse and play an important role in natural processes such as degradation of waste, fixation of carbon dioxide, and fermentation of organic matters, have not been thoroughly investigated for their antimicrobial production [8]. This might be due to the long-standing assumption that anaerobes were not capable of producing secondary metabolites such as polyketides and non-ribosomal peptides [9]. Perhaps, the pathogenicity of some members of the genus attracted more attention than the beneficial effects of non-pathogenic *Clostridium* species. Their metabolic diversity suggests they are of interest in exploring the potential for novel secondary metabolites possessing antimicrobial properties.

In recent years, the development of *in silico* strategies for the prediction of potential gene clusters, involved in the synthesis of bioactive compounds, has permitted a new understanding of the antimicrobial potential of various microorganisms from different environments. Genome sequencing and rapidly developing bioinformatics tools/methods provide opportunities to assess the biosynthetic potential of microorganisms prior to any biological testing or chemical analysis. Genome mining for the existence of biosynthetic pathways that allow microorganisms to synthesise secondary metabolites including antimicrobials has therefore become part of the emerging efforts to renew antimicrobial discovery. The genes responsible for the synthesis of secondary metabolites in bacteria are often organized as biosynthetic gene clusters (BGCs). These gene clusters usually consist of genes responsible for biosynthesis of precursors, assembly and modification of compound scaffolds, and genes required for regulation, export, and resistance [10]. BGCs are classified based on their products; ribosomally synthesized post -translationally modified peptides (RiPPs), non-ribosomal peptide synthetases (NRPSs), and polyketide synthases (PKSs). NRPSs and PKSs encode for modular enzymes that are involved in the biosynthesis of a large class of structurally diverse, clinically important bioactive compounds including antimicrobials [11].

Several computational tools including antiSMASH [12], ARTS [13], BAGEL 3 [14], Np.searcher [15], RODEO [16], and SMURF [17] have been developed to predict BGCs in bacterial and fungal genome sequences. Among them, antiSMASH (antibiotics and secondary metabolite analysis shell) has become a popular tool used widely to identify secondary metabolite BGCs in the genomes of interest [12]. This could be due to antiSMASH’s broad collection of tools and databases for genome mining, BGC analysis and domain analysis for a variety of secondary metabolites, and is a free and public webserver tool [10]. antiSMASH utilizes pHMMs (profile Hidden Markov Models) signature genes and PKS/NRPS signature domains for the detection of putative biosynthetic gene clusters in query genomes. Moreover, antiSMASH generated results are used by several other independent tools including ARTS (Antibiotic Resistance Target Seeker) and Big-SCAPE (BGC clustering and classification platform) [18].

Our previous study revealed the antimicrobial potential of Farm 4 soil conditioned medium originating from FS01, FS2.2, FS03, and FS04 *Clostridium* isolates [19]. In the present study, we investigated the under-explored biosynthetic potential of these *Clostridium* spp. for the synthesis of secondary metabolites belonging to antimicrobial compound groups and provided genome and culture-based evidence for their antimicrobial potential. Two isolates (FS01 and FS03) were sequenced in the current study and the other two isolates (FS2.2 and FS04) were previously sequenced [20, 21]. Culture dependent work demonstrated the antimicrobial properties of all four soil *Clostridium* isolates and the genome-based study revealed the presence of putative secondary metabolite gene clusters defining the biosynthetic capability of four soil *Clostridium* spp. to produce secondary metabolites, with potential antimicrobial properties.

## Results and discussion

### Growth inhibitory activity of four soil *Clostridium* isolates

**Fig. 1:**
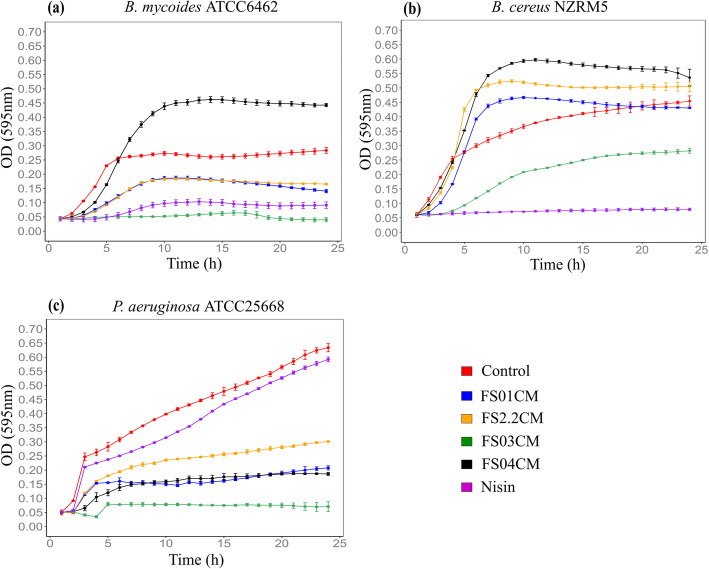
Effect of conditioned media derived from four soil *Clostridium* isolates on the growth of *B. mycoides* ATCC6462 (**a**), *B. cereus* NZRM5 (**b**) and *P. aeruginosa* ATCC25668 (**c**). Bacteria were grown in the presence of butterfield’s diluent (untreated control; red), FS01CM (FS01 conditioned media; blue), FS2.2CM (FS2.2 conditioned media; orange), FS03CM (FS03 conditioned media; green), FS04CM (FS04 conditioned media; black) and Nisin (purple) in the growth media (CMGS). Nisin (45 µM) and butterfield’s diluent served as positive and untreated control respectively. Error bars represent standard deviation (*n* = 3). The growth of *P. aeruginosa* was significantly reduced by all four CMs, while only FS03CM significantly inhibited the growth of *B. cereus* and there was a significant growth reduction of *B. mycoides* by FS01CM, FS2.2CM and FS03CM (*P* < 0.05 vs. untreated control)

In order to screen *Clostridium* isolates from soil for antimicrobial activity, conditioned media of all four *Clostridium* isolates were prepared after growing in cooked meat glucose starch medium (CMGS) as monocultures and tested for growth inhibition of *Bacillus mycoides* ATCC6462, *Bacillus cereus* NZRM5, and *Pseudomonas aeruginosa* ATCC25668. These bacteria were selected based on their adverse impact on food safety and quality, and human health. *B. cereus* has been reported to cause two types of food poisoning: diarrhoeal and emetic [22, 23]. *B. mycoides* is mainly associated with food spoilage and their food poisoning potential appears to be low [24]. Therefore, limiting the growth of these bacteria is important to maintain the safety and quality of food products. *P. aeruginosa* is a known opportunistic pathogen and a major cause of hospital acquired infections. Its control has become challenging due to resistance to multiple antimicrobial drugs [25]. *P. aeruginosa* has also been identified as a spoilage bacterium in dairy products [26]. Therefore, its control is important in the medical and food industries. FS01CM significantly inhibited the growth of *B. mycoides* and *P. aeruginosa*, but not *B. cereus* in comparison to untreated controls (*P* < 0.05). Similarly, FS2.2CM was active against *B. mycoides* and *P. aeruginosa*. Among all four isolates, the strongest and broadest antimicrobial activity was reported from FS03CM showing significant growth inhibition against all three test microorganisms compared to untreated controls (*P* < 0.05). FS04CM demonstrated the least activity by inhibiting only the growth of *P. aeruginosa* (Fig. 1). The positive control, nisin, showed strong antimicrobial activity against Gram-positive *Bacillus* spp., but not against Gram-negative *P. aeruginosa*, consistent with previous reports [27, 28]. In summary, conditioned media from all four *Clostridium* isolates showed various levels of antimicrobial activity against test microorganisms. Interestingly, all four CMs from the four *Clostridium* isolates produced stronger activity against *P. aeruginosa* than the widely used commercial food preservative nisin. However, as only a single growth condition was used to prepare the conditioned media from *Clostridium* isolates, this may not demonstrate their full antimicrobial potential. A single laboratory condition may not trigger the production of some specific metabolites in bacteria as their activation may require specific abiotic and/or biotic environmental cues [29].

### Genome sequencing and taxonomic characterization

The genomes of all four soil *Clostridium* isolates were sequenced using the Illumina MiSeq version 3 sequencing platform. The size of draft assembled genomes of the four *Clostridium* isolates ranged from 3.5 to 3.9 Mb in line with the size of previously reported clostridial genomes [30], and their respective genomic features are summarized in Table 1. Genome data of FS01, FS2.2, FS03, and FS04 isolates were submitted to NCBI under the accession number PRJNA705805, PRJNA642910, PRJNA706025, and PRJNA605262 respectively.

**Table 1:** General features of the genomes used in this study

	Isolate ID			
	**FS01**	**FS2.2**	**FS03**	**FS04**
Draft Genome size (bp)	3,529,942	3,620,293	3,953,295	3,984,260
Completeness (%)	98	97	83	98
G + C (mol%)	28	31	28	27
Genes (total)	3541	3614	3900	3640
CDSs (total)	3375	3489	3752	3483
Genes (coding)	3363	3428	3719	3430
rRNAs (5 s, 16 s, 23 s)	16, 25, 23	9, 16, 13	13 ,19, 18	11, 19, 16
tRNAs	98	83	94	107
Number of contigs	44	88	32	96
Contig N50	1,993,941	106,458	730,680	91,086

A preliminary identification of four soil isolates was carried out using PCR based 16 S rRNA gene sequence analysis as described previously [19]. This indicated that FS01, FS2.2, FS03, and FS04 were taxonomically closely related to *Paraclostridium bifermentans* (98.1 %), *Clostridium cadaveris* (100 %), *Terrisporobacter glycolicus* (95.6 %), and *Clostridium senegalense* (98.2 %) respectively. In the current study, whole genome sequences of these four soil *Clostridium* isolates were used to further confirm the taxonomic assignment and the functional relationships between the isolates and closely related microorganisms.

**Fig. 2:**
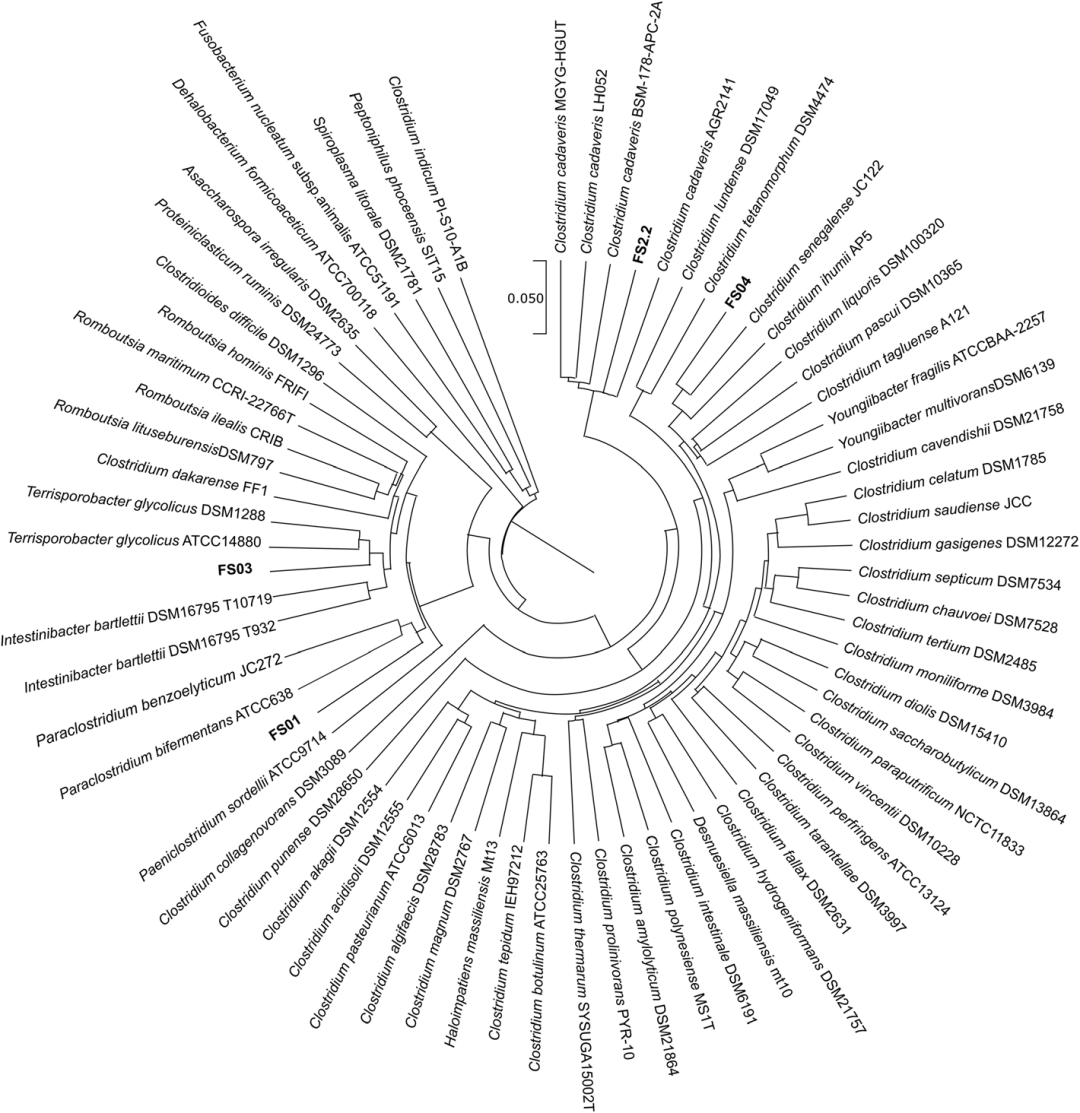
16 S rRNA-based phylogenetic tree inferred with FastME 2.1.6.1 from GBDP distances calculated from 16 S rRNA gene sequences. The branch lengths are scaled in terms of GBDP distance formula d_5_. Four soil *Clostridium* isolates are highlighted in bold

A phylogenetic tree was constructed using extracted the 16 S rRNA gene sequences from whole genome sequences and indicated that FS01 clustered together with *Paraclostridium bifermentans* and *Paraclostridium benzolyticum*; FS2.2 was closely related to *Clostridium cadaveris*; FS03 was closely related to *Terrisporobacter glycolicus*, and FS04 was closely related to *Clostridium senegalence* (Fig. 2). These phylogenetic neighbours were similar to those previously reported by PCR based 16 S rRNA gene sequence results from all four isolates. *C. cadaveris* genomes were manually added to the TYGS analysis due to the absence of any draft genome sequence of its type strains in the TYGS database.

Genome-based methods including *in silico* digital DNA-DNA hybridization (dDDH) and average nucleotide identity (ANI) have been identified as important criteria in microbial species delineation [31, 32]. These values have been coined as the overall genome related index (OGRI) [33]. Digital DNA:DNA hybridization values for each genome of the *Clostridium* isolates and their closest neighbours are shown in Table 2. ANI values, which can be used as a measure of genetic relatedness between genomes, were also calculated for the same genome pairs [31]. dDDH values and ANI values obtained for FS01 and *Paraclostridium bifermentans* (formerly known as *Clostridium bifermentans*), FS2.2 and *Clostridium cadaveris*, FS04 and *Clostridium senegalense* were over the recommended species boundary cut-off values of 70 % dDDH and 95 % ANI, respectively [31, 34]. These results indicate that these three isolates belong to the same species they have been compared with (Table 2). dDDH values (66.8 %) and ANI values (< 95 %) obtained between FS03 and *Terrisporobacter glycolicus* ATCC 638, and FS03 and *Terrisporobacter glycolicus* DSM 1288 were below species cut-off values. Therefore, FS03 does not belong to the species *T. glycolicus* (formerly known as *Clostridium glycolicum*) even though it is the closest relative according to 16 S rRNA based phylogenetic analysis. These results indicate that FS03 may represent a novel species of the genus *Terrisporobacter*.

**Table 2:** Intergenomic digital DNA-DNA hybridization and average nucleotide identity values between *Clostridium* isolates and their closest phylogenetic neighbours

Isolate	Subject strain	dDDH (CI) in %	Two-way ANI (%)
FS01	*Paraclostridium bifermentans* ATCC638	83.2 (79.9–86.1)	96.24
FS2.2	*Clostridium cadaveris* AGR2141	90.2 (87-92.7)	99.61
FS03	*Terrisporobacter glycolicus* ATCC14880	66.8 (63.4–70.0)	92.48
	*Terrisporobacter glycolicus* DSM1288	66.8 (63.4–70.1)	92.52
FS04	*Clostridium senegalense* JC122	86.6 (83.5–89.2)	95.38

*CI* confidence intervals; *ANI *average nucleotide identity; *dDDH* digital DNA-DNA hybridization

**Fig. 3:**
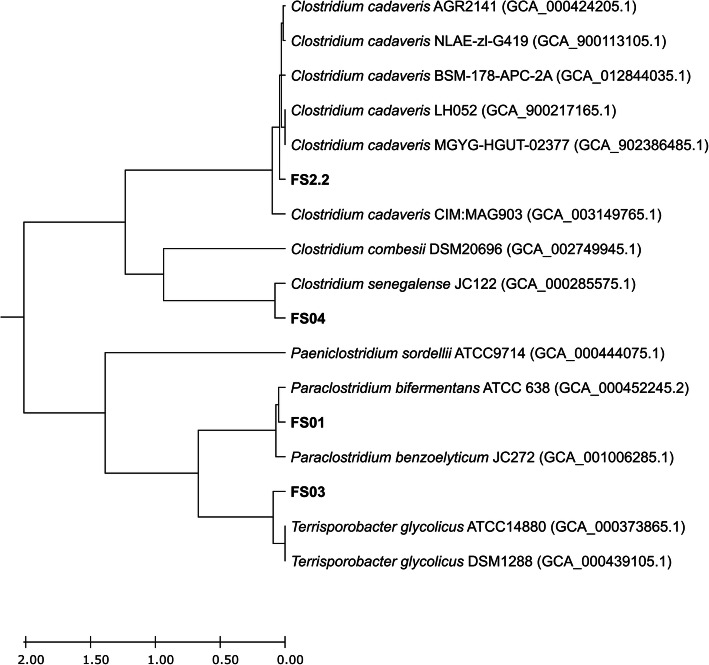
Inferred phylogenetic tree based on the functional genome distribution (FGD). All the genomes were subjected to FGD analysis, and the resulting distance matrix was imported into MEGA X version 10.2.2. and visualized using unweighted pair group method with arithmetic mean (UPGMA) method

A comparative whole genome analysis of four soil isolates and closely related *Clostridium* spp. was conducted by computing the functional genome distribution (FGD). FGD analysis computes the overall levels of microbial genome similarities by comparing amino acid sequences predicted from each bacterial open reading frames (ORFeomes) [35]. The FGD comparative genomics approach carries out genome to genome comparisons highlighting functional associations rather than evolutionary relationships. Based on this analysis, FS01, FS2.2, and FS04 isolates are functionally closely related to their previously assigned species, *Paraclostridium bifermentans*, *Clostridium cadaveris*, and *Clostridium senegalence* respectively (Fig. 3). FS03 showed a close functional relationship to *Terrisporobacter glycolicus* similar to its TYGS phylogenetic analysis results. However, OGRI suggested that FS03 as a novel species, different from *Terrisporobacter glycolicus.*

### Detection of putative virulence factors (VFs)

Bacteria exhibiting antagonistic activity against other harmful microorganisms are often considered for developing probiotics. When considering bacteria to be used as probiotic, their pathogenicity is of the utmost important. An array of animal and microbiological assays is available to demonstrate the safety of potential probiotic strains. Nowadays, the evaluation of genome sequences has become a vital part of a thorough safety evaluation [36, 37].

The genus *Clostridium* includes important human and animal pathogens and some of the members produce exotoxins responsible for diseases such as those causing botulism, tetanus, and gas gangrene. *Clostridium perfringens* and *Clostridium botulinum* are two main toxin producers causing foodborne illnesses [38]. In the present study, soil *Clostridium* isolates shown to possess antimicrobial properties were evaluated for the presence of virulence genes using their whole genome sequences.

The presence of putative virulence genes in all four *Clostridium* genomes was investigated using VFanalyzer. VFanalyzer is a virulence factor data base (VFDB) integrated automatic pipeline, which can identify known or potential virulence factors in complete or draft genomes of bacteria [39]. The VFanalyser predicted three putative *Clostridium* toxin related genes; *plc* (alpha-toxin), *colA* (kappa-toxin), and *pfoA* (perfringolysin O) in the FS01 genome and one putative toxin related gene; *nagH* (Mu-toxin) in the FS2.2 genome. FS03 and FS04 genomes were not found to harbour putative genes for the main *Clostridium* toxins including alpha-clostripain (*cloSI*), alpha-toxin (*pIc*), beta2 toxin (*cpb2*), *Botulinum* neurotoxin (*atx*), *C. novyi* alpha-toxin (*tcnA*), *C. perfringens* enterotoxin (*cpe*), *Clostridium difficile* toxin (*cdtA*, *cdtB*), enterotoxin (*entA*, *entB*, *entC*, *entD*), kappa-toxin (*colA*), mu-toxin (*nagH*, *nagI*, *nagJ*, *nagK*, *nagL*), perfringolysin O (*pfoA*), sialidase (*nanH*, *nanI*, *nanJ*), tetanus toxin (*tetX*), toxin A (*toxA*), and toxin B (*toxB*). Based on these results, FS03 and FS04 isolates could be more suitable for developing probiotics as genes for virulence factors were absent. However, the expression of *plc*, *colA*, and *pfoA* toxin genes detected in FS01 isolate, are usually regulated by the VirS/VirR two component regulatory system [40] and genes responsible for this regulatory system (*VirS* and *VirR*) were not found in FS01 genome. However, further assessments are required to confirm their safety to be used as probiotics.

### Identification of biosynthetic gene clusters (BGCs)

In recent years, strict anaerobes such as *Clostridium* spp. have started to gain attention as potent antimicrobial producers with the advent of computational genomics [29]. Recent studies have focused on obtaining genomic information on the secondary metabolism of anaerobic bacteria including *Clostridium* species [41–43]. In the current study, four soil *Clostridium* isolates demonstrated antimicrobial activity against significant food and human associated bacteria and were further investigated for their genetic potential to produce various secondary metabolites, using their assembled whole genome sequences and antiSMASH 5.0 genome mining pipeline.

**Fig. 4:**
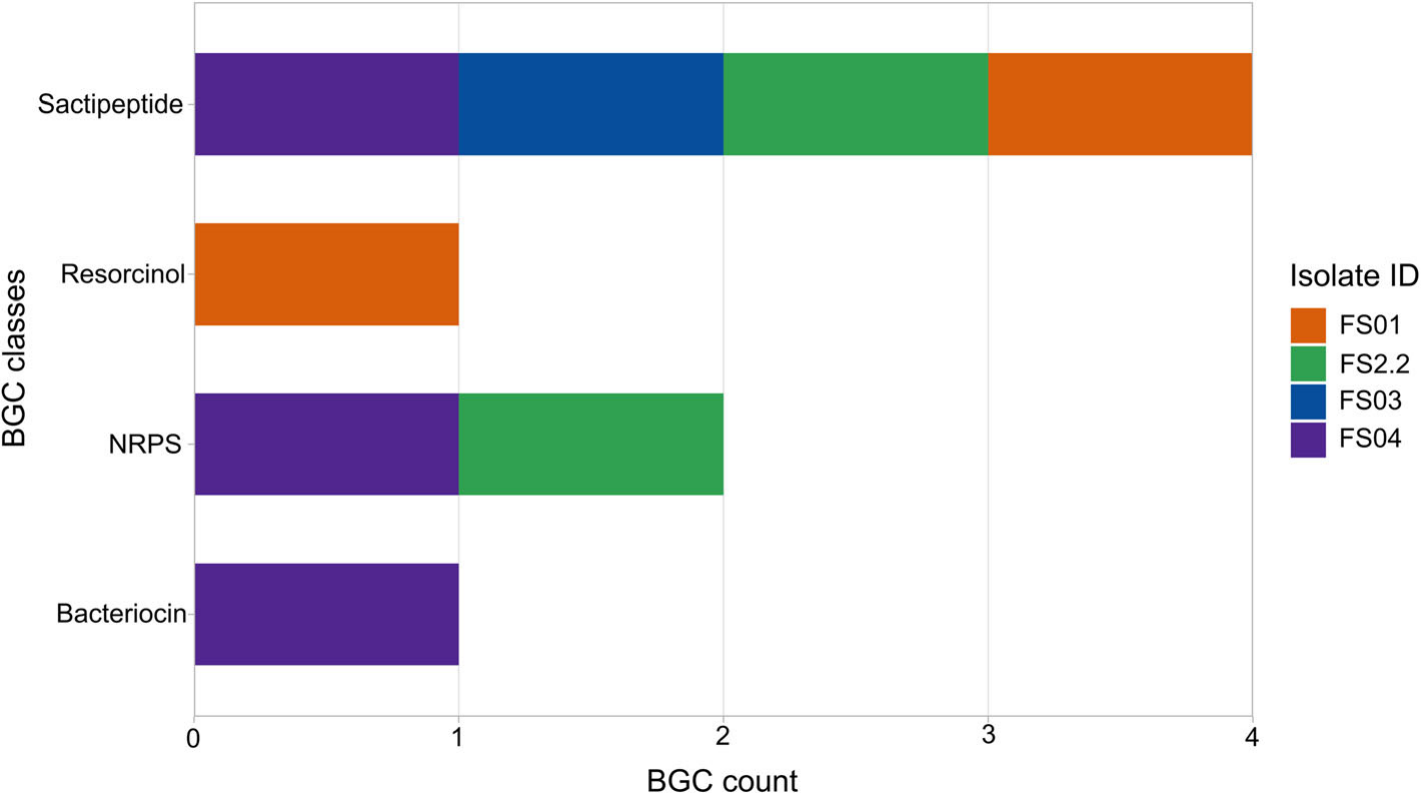
Total number of different BGCs (BGC count) predicted in all four *Clostridium* isolates. Different colours depict in which *Clostridium* isolate they were detected

antiSMASH analysis predicted a total of 8 potential secondary metabolite biosynthetic gene clusters in all four *Clostridium* genomes (Fig. 4). FS04 showed the highest BGC count detecting one sactipeptide, one NRPS, and one bacteriocin gene clusters in its genome. FS03 harboured only one BGC, which encoded for a sactipeptide, while the other two genomes (FS01 and FS2.2) featured two BGCs each as shown in Fig. 4. None of the detected clusters could be assigned to a known compound (Table 3). Therefore, it is likely that all of the metabolites synthesised by the detected BGCs have not been fully characterized.

**Table 3:** Details of predicted putative BGCs in all four *Clostridium* isolates by antiSMASH 5.0

Isolate	BGC type	Location (nt)		Most similar known cluster
		**From**	**To**	
FS01	Resorcinol	157,419	198,534	NA
	Sactipeptide	149,074	169,217	NA
FS2.2	NRPS	45,623	88,681	NA
	Sactipeptide	4,427	24,633	NA
FS03	Sactipeptide	143,920	164,063	NA
FS04	Sactipeptide	63,762	83,899	NA
	Bacteriocin	1	10,155	NA
	NRPS	41,090	83,957	NA

*NA* not available

### RiPPs gene clusters (sactipeptides and other bacteriocins)

**Fig. 5:**
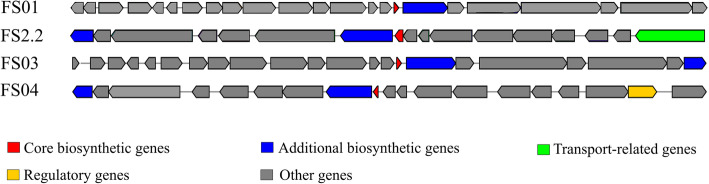
Detected putative sactipeptide gene clusters in soil *Clostridium* isolates

The most abundant BGC type annotated in the genomes of the four bacterial isolates was sactipeptide (Fig. 5). Sactipeptides (Sulfur-to-alpha carbon thioether crosslinked peptides) are a class of ribosomally synthesized post-translationally modified peptides (RiPPs). The unique feature of these compounds is the assembling of precursor peptides from encoded genes followed by maturation to the relevant core peptide by introducing at least a one intramolecular thioether bridge, connecting cysteine sulphurs with an unreactive α-carbon of the partnering amino acid [44, 45]. The first reported sactipeptide is subtilosin A from *Bacillus subtilis* [46]. Since then, more members of this class have been identified from *Bacillus* species. In recent years, genome mining of various bacterial genomes has revealed the presence of putative sactipeptide biosynthesis gene clusters in other microorganisms including *Clostridium* spp. [43]. It is postulated that during the maturation process, thioether bond formation is catalysed by a radical *S*-adenosylmethionine (SAM) enzyme encoded by the relevant biosynthetic gene cluster [47]. antiSMASH predicted the core biosynthetic genes of all four sactipeptide clusters using the TIGR03973 HMM profile. Members of TIGR03973 are designated as “Six Cysteines in Forty-Five residues” (SCIFF) and are predicted ribosomal product precursors linked with an uncharacterized radical SAM protein [48]. The antiSMASH analysis showed that core peptides of predicted sactipeptides were SCIFF belonging to the genus *Clostridium* and were not associated with any known sactipeptides.

The antiSMASH ‘ClusterBlast’ tool compares the similarity of individual genes and their arrangement in the query cluster with a comprehensive database of predicted BGCs of publicly available genomes. This comparison identifies other microorganisms harbouring similar BGCs as the query cluster [10]. Furthermore, the ‘KnownClusterBlast’ module compares any predicted BGCs with the known/ characterized BGCs in the Minimum Information about a Biosynthetic Gene cluster (MIBiG) database to identify the closest compound/product [49]. The ClusterBlast results indicated that several other *Paraclostridium bifermentans* strains and *Paeniclostridium sordellii* strains harbour 100 % identical gene clusters to the predicted FS01 sactipeptide gene cluster in this study (Additional file 1: Fig. S1a). antiSMASH annotated sactipeptide clusters in FS2.2 and FS03 genomes were also found to have 100 % similar gene clusters in *Clostridium cadaveris* and *Terrisporobacter glycolicus* strains, respectively. No other bacteria were found to have 100 % identical gene clusters to the FS04 sactipeptide cluster. The best match was only 94 % gene cluster similarity with *Clostridium senegalense*. However, only a single gene is potentially different between two gene clusters (Additional file 1: Fig. S1). Since the non-identical gene is located at the margin of the gene cluster and annotated as an ‘other’ gene, it may not be involved in the sactipeptide biosynthesis. Accordingly, FS04 and *Cl. senegalense* sactipeptide clusters would be 100 % identical in terms of functionality. Sactipeptide clusters predicted in all four *Clostridium* genomes showed no similarity to a BGC encoding for a known sactipeptide. This is not surprising as only one sactipeptide cluster, thuricin CD from *Bacillus thuringiensis*, has been characterized and included in the MIBiG database so far [49]. To date, no sactipeptide has been chemically identified and isolated from *Clostridium* spp. [29]. Therefore, these results encourage further studies to identify and characterize sactipeptides from *Clostridium* species.

**Fig. 6:**
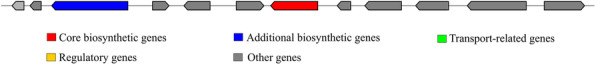
A bacteriocin gene cluster detected in the FS04 genome

Another bacteriocin/unspecified RiPP gene cluster was detected in FS04 (Fig. 6). This gene cluster contains a core gene encoding a protein harbouring a DUF692 domain. Members of the DUF692 protein family are involved in bacteriocin production [50]. An additional biosynthetic gene encoding for a putative peptidase was also annotated in the same gene cluster. There was no other regulatory, transport-related or resistance related gene annotated in the cluster. Cluster blast analysis of the bacteriocin gene cluster did not find a 100 % identical gene cluster in any other bacteria in the antiSMASH database. The bacteriocin cluster showed no relatedness to any known compounds and the most similar cluster was found in the *Clostridium senegalense* JC122 strain, which showed only 75 % similarity to the FS04 bacteriocin cluster (Additional file 1: Fig. S2).

We cannot further elaborate on the putative functions of these RiPPs as they did not show any relatedness to known compounds. This could be due to the lack of information as only three RiPP clusters from *Clostridium* spp. have been characterised in the MIBiG database [49]. However, these results expand the knowledge regarding the presence/abundance of potentially novel RiPP gene clusters among species of the genus *Clostridium*. Further studies are required to understand their products and functions.

### NRPS gene clusters

Microbial products of non-ribosomal peptide (NRP) origin have been significant bioactive compounds possessing antimicrobial, antifungal, anti-tumour properties, and can be used as immunosuppressants [11]. NRPs are synthesized by dedicated non-ribosomal peptide synthetases (NRPSs), which are modular multi-domain enzyme complexes, that serve as templates and biosynthetic machinery for non-ribosomal peptide synthesis [51–53]. NRPSs assemble non-ribosomal peptides through a series of repeating steps that are catalysed by synchronized actions of three catalytic domains: The adenylation domain (A), thiolation or peptidyl carrier protein domain (PCP), and the condensation domain (C) [54]. These catalytic domains when organized together comprise a minimal NRPS module, which is responsible for the incorporation of a single residue into the final peptide product. A fourth domain, a thioesterase, catalyses the release of the peptide from the NRPS [52].

**Fig. 7:**
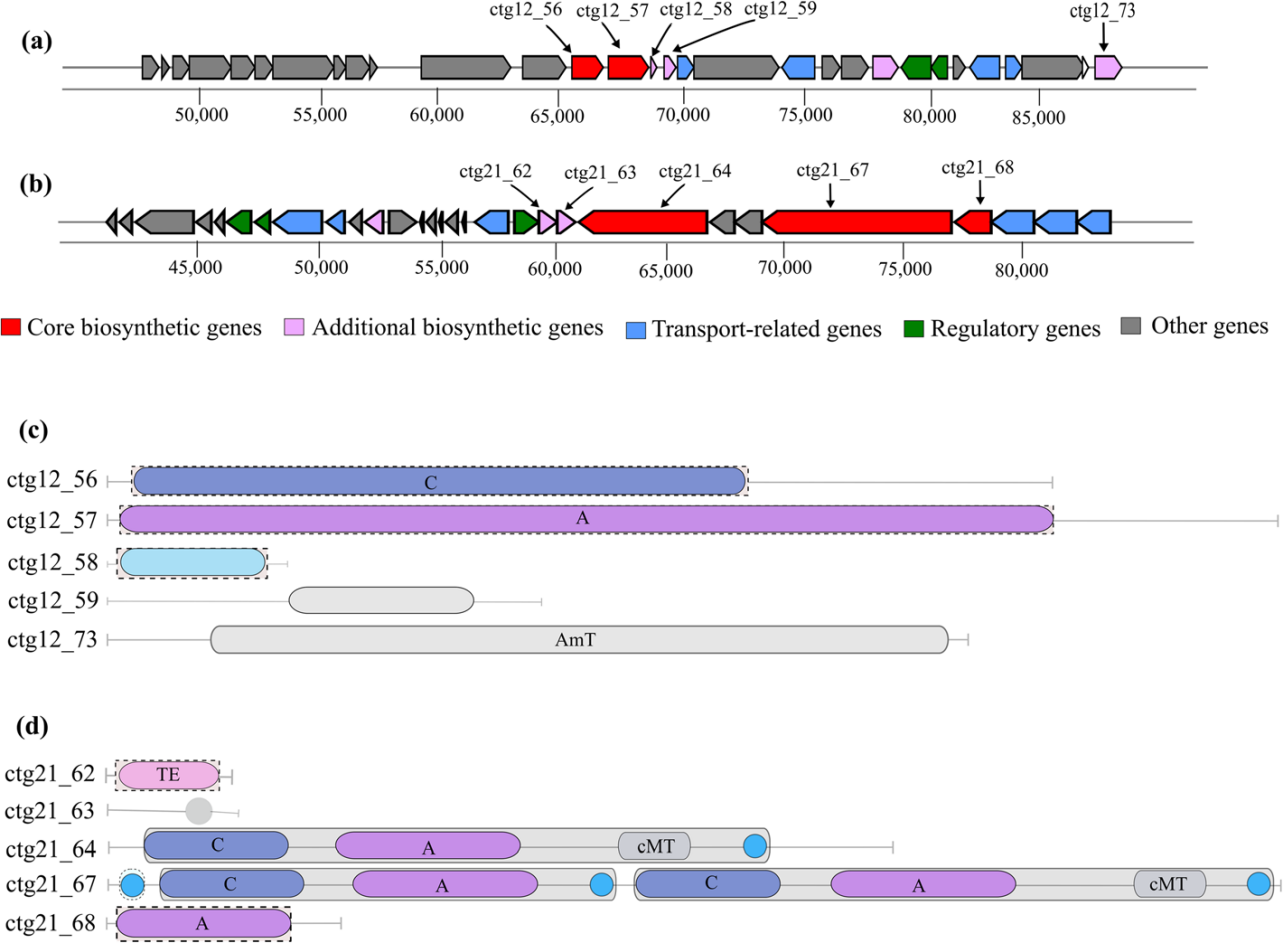
The predicted NRPS gene clusters. (**a**) NRPS gene cluster predicted in the FS2.2 genome. (**b**) NRPS gene cluster detected in the FS04 genome. (**c**) Module and domain annotation of the FS2.2 NRPS gene cluster. (**d**) Module and domain annotation of the FS04 NRPS gene cluster. Gene locus tags are given on the left side of the domain architecture and in the corresponding genes of the gene cluster

antiSMASH analysis of the four *Clostridium* isolates in the present study predicted the presence of two putative NRPS gene clusters in the genomes of FS2.2 and FS04. These two gene clusters and their domain organization were found to be different to each other (Fig. 7). Notably, both clusters shared no similarity with those of previously characterized known antimicrobial compounds in the MIBiG database. Furthermore, they appear to be unique NRPS clusters due to the absence of identical clusters in closely related microorganisms. According to cluster blast results, the FS2.2 cluster found the best match with the *Clostridium cadaveris* AGR2141 genome with 64 % cluster similarity. The NRPS cluster detected in FS04 showed the highest similarity of 55 % to a NRPS cluster in the genome of *Clostridium senegalense* JC122. These results demonstrate that the predicted clusters may encode producer specific novel NRPS compounds.

In recent years, several studies have predicted the secondary metabolite synthesis potential of anaerobic bacteria including some *Clostridium* species. A Letzel, SJ Pidot and C Hertweck [43] investigated published genomes of anaerobic bacteria including some *Clostridium* spp. for the occurrence of RiPP encoding gene clusters and found that over 25 % of the anaerobic genomes tested possessed the genetic basis for producing RiPPs. Another study evaluated 211 published anaerobic genomes in search of specifically putative NRPS and PKS and revealed that 33 % of test genomes possessed PKS and NRPS genes [42]. S Behnken and C Hertweck [41] investigated the presence of PKS gene clusters in the genomes of several *Clostridium* spp. and found a wide distribution of putative PKS gene clusters only in non-pathogenic strains. However, to date, very few antimicrobial secondary metabolites have been identified and characterised from *Clostridium* species. Therefore, there is limited information on the biosynthetic gene clusters associated with known clostridial antimicrobials limiting the prediction capabilities. In the present study, in agreement with the antagonistic activities, all four *Clostridium* isolates were predicted to have at least one or more biosynthetic gene clusters in their genomes. All putative gene clusters identified in the present study were not associated with any currently known clostridial or other secondary metabolites in the MiBiG database, indicating that they might encode for novel compounds. Furthermore, some of the putative clusters were unique to the *Clostridium* isolates used in this study suggesting their respective novelty to produce putative secondary metabolites.

However, it should be taken into consideration that neither the genome-based nor the culture-based approach alone can show the full antimicrobial potential of microorganisms due to their own limitations. Genome mining techniques including antiSMASH are not able to exactly predict the full capacity of a bacterium to produce bioactive compounds, including antimicrobials. Bioinformatic tools such as antiSMASH use BGC prediction algorithms, which are based on the identification of known biosynthetic pathways to some extent and it can be speculated that true novel biosynthetic pathways may not be detected. Most of the genome mining tools use conserved domains of biosynthetic enzymes or pHMMs (profile Hidden Markov Models) signature genes for identification of core genes of a biosynthetic pathway. This implies a limitation of these tools not being able to identify novel pathways and enzymes. To improve the BGC detection, antiSMASH has implemented the ‘ClusterFinder’ algorithm to identify BGCs that are not detected by the rule-based genome mining. This technique still has some bias towards currently known pathways as BGC detection has been trained using source data from known pathways [10]. The assembly quality of the query genomes is important to achieve reliable results from antiSMASH as its genome analysis relies on identifying genes from the coding regions [10]. On the other hand, the prediction of the presence of a certain biosynthetic pathway does not fully ensure the production of associated secondary metabolites. Biosynthetic gene clusters could be either silent or active under specific growth conditions. Activation of gene clusters may require specific external factors such as presence/density of other microbes or (lack of) nutrients [55]. However, the limitation in detecting silent metabolic pathways by conventional culture-based methods has been overcome by genome mining. Furthermore, anaerobic bacteria such as *Clostridium* spp. can utilize a wide range of substrates for their anaerobic fermentation and produce various metabolites based on the substrates used [56]. Some of these small metabolites produced from anaerobic fermentation other than metabolites synthesized as a direct regulation of gene clusters, may also possess antimicrobial activities. For instance, *Clostridium difficile* was reported to produce *para-*cresol through the fermentation of tyrosine, which showed antagonistic activity against certain bacteria [57]. Despite the above limitations, genome mining has been shown to assist antimicrobial discovery by providing valuable insight into the genomic potential to produce bioactive secondary metabolites.

## Conclusions

BGCs encoding for uncharacterized RiPPs and NRPSs were identified from four soil *Clostridium* isolates, that showed antimicrobial activities against bacteria associated with human health, food quality and safety, predicting their genetic potential to produce novel secondary metabolites. This knowledge serves as a basis for future investigations to identify and characterize potent antimicrobials from *Clostridium* isolates and also expands the current knowledgebase on the potential of the genus *Clostridium* to produce bioactive compounds.

## Methods

### Bacterial strains and growth conditions

All four bacterial isolates (FS01, FS2.2, FS03, and FS04) used in this study were previously isolated from soil collected from bovine dairy farms (Manawatu region in New Zealand) and identified as *Clostridium* spp. by PCR based 16 S rRNA gene sequence analysis [19]. Frozen glycerol stocks of these four soil *Clostridium* isolates were revived on sheep blood agar (SBA) plates following incubation at 35 °C overnight under anaerobic conditions. Each isolate grown on SBA plates was then used to inoculate either cooked meat glucose starch medium or tryptic soy broth (TSB). *Bacillus mycoides* ATCC 6462, *Bacillus cereus* NZRM 5, and *Pseudomonas aeruginosa* ATCC 25,668 were obtained from Environmental Science and Research (ESR), New Zealand. They were grown in tryptic soy broth (TSB) and incubated at 35 ^o^C overnight for growth inhibition assays.

### Preparation of conditioned medium from soil *Clostridium* isolates

*Clostridium* enriched conditioned media were prepared from all four individual isolates following a previously described method with some modifications [19]. Briefly, each *Clostridium* isolate grown on SBA plates was used to inoculate cooked meat glucose starch medium (45 mL) supplemented with yeast extract (0.0005 %), hemin (0.1 %), and vitamin K (1 %) and incubated in a 35 ^o^C anaerobic chamber (Don Whitley Scientific, United Kingdom) for 48 h. After the incubation, enriched media were centrifuged at 10,000 × g for 40 min at 4 ^o^C and supernatants were filter-sterilized using 0.22 μm polyvinylidene fluoride syringe filters (Millipore, Ireland). Sterile conditioned media were aliquoted and stored frozen at -20 ^o^C until use. Conditioned media prepared from FS01, FS2.2, FS03, and FS04 isolates were denoted as FS01CM, FS2.2CM, FS03CM, and FS04CM respectively.

### Growth inhibition assay

Microplate turbidimetric growth inhibition assays were conducted according to a method described previously [19]. Briefly, an overnight grown bacterial culture was adjusted to 1 × 10^7^ CFU/mL cell density and 50 µL were mixed with CMGS broth (50 µL) and conditioned medium (100 µL) in a 96-well flat bottom microtiter plate (Thermo scientific, Denmark) and covered with a sealing membrane (Diversified Biotech, USA). Bacterial growth of the test cultures in each well was assessed by incubating the plate in a microplate spectrometer at 35 ^o^C and measuring the optical density (OD) at 595 nm for 24 h. Nisin (45 µM) (Sigma-Aldrich, USA) and Butterfield’s diluent were used as the positive control and untreated control respectively. Corresponding blanks (growth medium, conditioned media, and nisin) were used for background correction.

All experiments were performed in triplicate and data were presented in mean ± standard deviation. The area under the experimental growth curve (AUC), which represents the overall bacterial growth under given growth conditions was calculated using the R package ‘growthcurver’ [58]. Single factor Analysis of Variance (ANOVA) was performed to check the statistical significance of CM treatments on the bacterial growth by comparing the growth in the presence and absence of CM using AUC values. The values with *P* < 0.05 were considered statistically significant.

### DNA extraction and purification

Genomic DNA from pure cultures grown in TSB was extracted by using a phenol-chloroform extraction method as described earlier [59] with minor modifications. Bacterial cultures (5 mL) were centrifuged at 10,000 × g for 2 min and the cell pellets were resuspended in 1 mL TE buffer (composed of 10 mM Tris-HCl and 1 mM EDTA, pH 08) (Thermo Fisher Scientific, Lithuania). Resulting cell suspensions were washed twice with TE buffer by centrifuging at 10,000 × g for 2 min. After washing, harvested cell pellets were resuspended in 200 µL genomic DNA solution composed of 1 M sucrose (Fluka, Germany) in TE buffer. Fifty microliters of freshly prepared lysozyme (50 mg/mL) (Sigma-Aldrich, USA) were then added to the cell suspensions and the resulting mixtures were incubated at 37 ^o^C for 30 min. Following the incubation, 100 µL of 20 % (w/v) sarkosyl (sodium lauroyl sarcosinate) (Sigma-Aldrich, UK) and 15 µL of RNase A (DNase free, 10 mg/mL) (Sigma-Aldrich, UK) were added and incubated at 37 ^o^C for another 30 min. Then, 7.5 µL of proteinase K (10 mg/mL) (Ambion, USA) were added and incubated at 37 ^o^C for 30 min. The lysate extraction was carried out three times with 600 µL of phenol/chloroform/isoamyl alcohol (25:24:1) ) (Sigma-Aldrich, USA) and centrifuged at 20,000 × g for 20 min. The DNA was precipitated using 50 µL of 3 M sodium acetate (pH 5.2) (BDH, UK) and three volumes of ice-cold absolute ethanol at -20 ^o^C for overnight, followed by a washing step with 750 µL of 70 % ethanol and centrifugation at 17,000 × g for 5 min. After removing supernatants, genomic DNA pellets were air-dried for about 15 min and re-suspended in 100 µL of sterile DNase and RNase free water. All samples were stored at 4 ^o^C overnight, they were separated by 0.8 % gel electrophoresis, and visualized by Gel Doc XR system (Bio-Rad Laboratories, USA). DNA quality and quantity were assessed using Qubit 4 fluorometer (Thermo Fisher Scientific Inc., USA) and NanoDrop 1000 spectrophotometer (Thermo Fisher Scientific Inc., USA).

### Whole genome sequencing, assembly, and annotation

High quality genomic DNA extracted from four *Clostridium* isolates was sequenced using Illumina MiSeq version 3 sequencing platform at the Massey University Genome Services, New Zealand. DNA library preparation was performed with the Celero PCR workflow with an enzymatic fragmentation DNA-seq library preparation kit (NuGEN, USA). Quality and quantity assessments of DNA libraries were carried out as a quality control check before sequencing. The resulting reads were quality trimmed, filtered, and *de novo* assembled with the A5-miseq assembler (version 20,160,825) under the default settings [60]. The genome completeness was assessed using BUSCO (version 1.22) [61].

### Phylogenetic and functional genome distribution (FGD) analyses

A 16 S rRNA gene sequence based phylogenetic tree was constructed using the Type (Strain) Genome Server (TYGS) webserver [62]. Briefly, draft genomes of FS01, FS2.2, FS03, and FS04 together with four whole genomes of *C. cadaveris* strains from the NCBI database (GCA_000424205.1, GCA_900217165.1, GCA_902386485.1 and GCA_012844035.1) were submitted to the TYGS webserver. TYGS extracted available 16 S rRNA gene sequences from query genomes using RNAmmer [63] and then performed NCBI BLAST + analyses using all possible pairs of 16 S rRNA gene sequences in the TYGS database. The genomes with the top 50 BLAST bitscores were used for the calculation of the Genome BLAST Distance Phylogeny (GBDP) values. GBDP values were calculated between the selected 16 S rRNA gene sequences under the algorithm ‘coverage’ and distance formula *d*_*5*_ [64], and the closest relatives were defined as the genomes with the lowest ten 16 S rRNA gene GBDP distances between each query genome and type strains. The resulting GBDP distances were used to construct a 16 S rRNA gene sequence based phylogenetic tree with FastME 2.1.6.1 including subtree pruning and regrafting (SPR) post processing [65]. The inferred tree was visualized using MEGA X version 10.2.2 [66].

The digital DNA:DNA hybridization (dDDH) values of each of the *Clostridium* genome and their closest neighbours were computed using Genome-to-Genome Distance Calculator (GGDC version 2.1) in TYGS analysis tool with default parameters [62, 64]. The average nucleotide identity (ANI) values between each *Clostridium* genome and their closest neighbours were calculated using the ANI calculator developed by Kostas lab with default parameters (http://enve-omics.ce.gatech.edu/ani/). ANI values were calculated as the mean identity of BLASTN matches [31].

Functional genome distribution (FGD) analysis investigates the genomic differences between bacteria and interprets the genetic diversity [35]. FGD analysis was carried out using all four *Clostridium* isolates and their closest neighbours identified from TYGS phylogenetic analysis. All the genomes required for the analysis except the four *Clostridium* isolates were downloaded in FASTA format from NCBI database. They were concatenated using a universal spacer-stop-spacer sequence and automatically annotated using GAMOLA 2 software package [67]. FGD analysis was performed using the predicted open reading frames (ORFeomes) of all genomes. The resulting distance value matrix was imported into MEGA X version 10.2.2 and visualized using an unweighted pair group method with arithmetic mean (UPGMA) [66].

### Analysis of putative biosynthetic gene clusters (BGCs) and virulence genes

Biosynthetic gene clusters encoding for secondary metabolites were predicted and annotated using the web server version of antiSMASH 5.0 [12] with default parameters and in combination with the ClusterFinder algorithm, which is a hidden Markov model based probabilistic algorithm to detect BGCs of both known and unknown groups [68]. antiSMASH ClusterBlast and KnownClasterBlast modules were used for comparing detected gene clusters to other sequenced genomes and known gene clusters. The web tool VFanalyzer was used to identify known/potential virulence factors in the genomes of four *Clostridium* isolates [39].

## Supplementary Information

**Additional file 1.**

## Data Availability

Data from the following public databases was used for the analysis. •Draft genomes from the national (NCBI) assembly database. https://www.ncbi.nlm.nih.gov/assembly/. •antiSMASH database. https://antismash-db.secondarymetabolites.org/. •Type (Strain) Genome Server (TYGS) database. https://tygs.dsmz.de/. Draft whole genome sequences of FS01, FS2.2, FS03 and FS04 isolates were submitted to NCBI under the accession number PRJNA705805, PRJNA642910, PRJNA706025 and PRJNA605262, respectively.

## Abbreviations

dDDH: DNA-DNA hybridization
ANI: average nucleotide identity
FGD: functional genome distribution
BGCs: biosynthetic gene clusters
RiPPs: ribosomally synthesized post-translationally modified peptides
NRPs: non-ribosomal peptides
NRPSs: non-ribosomal peptide synthetases
PKSs: polyketide synthases
pHMMs: profile hidden markov models
Big-SCAPE: BCG clustering and classification platform
PCR: Polymerase chain reaction
TSB: Tryptic soy broth
CMGS: Cooked meat glucose starch medium
OD: Optical density
TYGS: Type (Strain) genome server
