# Supplementary material for: Culture and genome-based analysis of four soil Clostridium isolates reveal their potential for antimicrobial production
Source: BMC Genomics. 2021 Sep 22;22:686. doi: 10.1186/s12864-021-08005-2 (PMC8456703; doi:10.1186/s12864-021-08005-2)
Supplement: Supplementary file 1 — Additional file 1. [file 12864_2021_8005_MOESM1_ESM.docx]

**a**


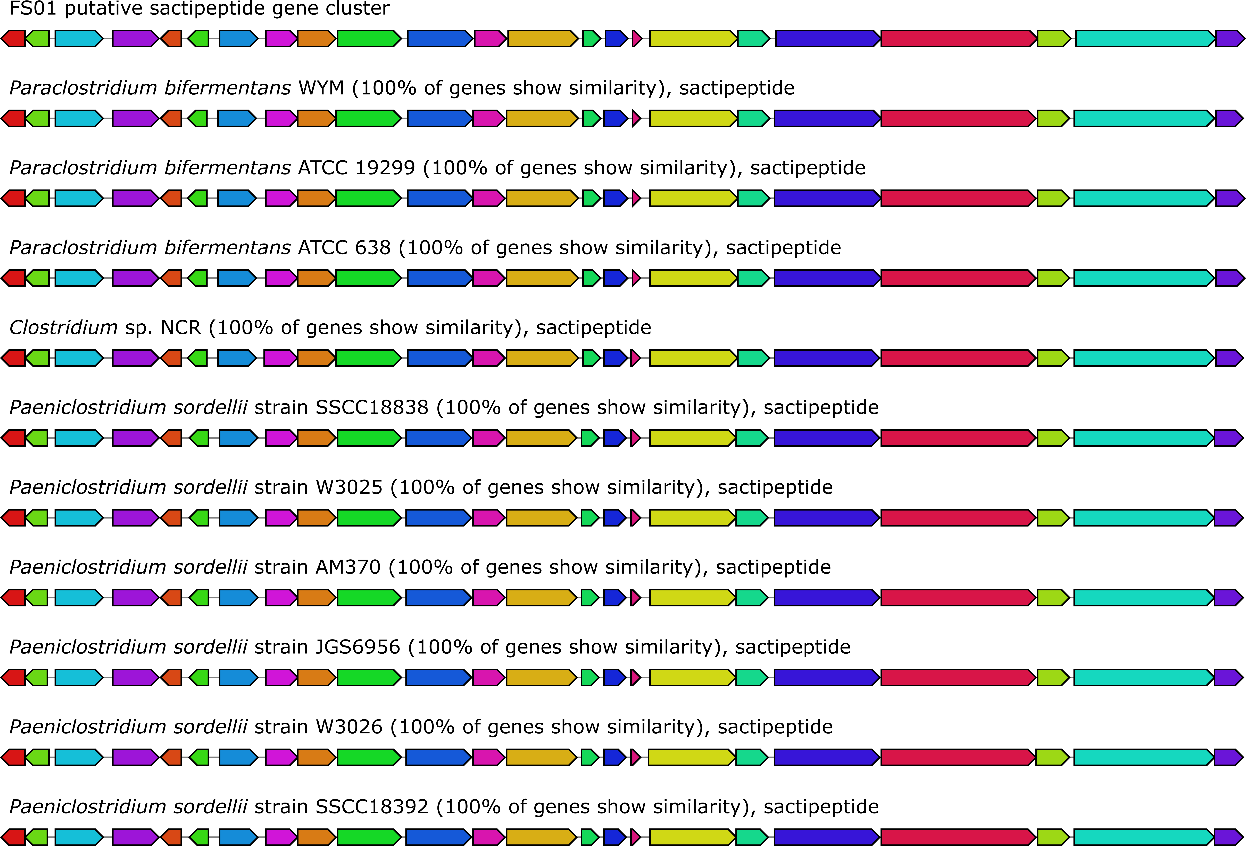

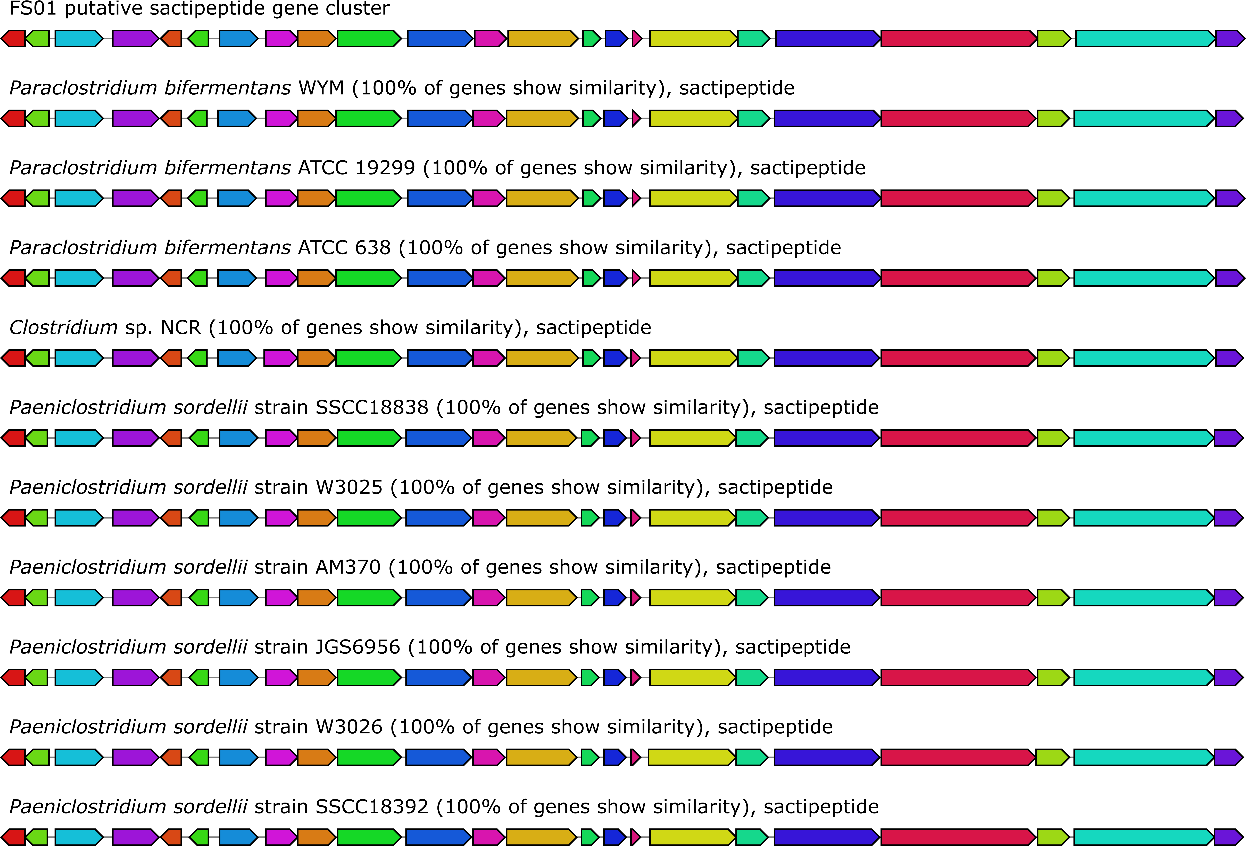


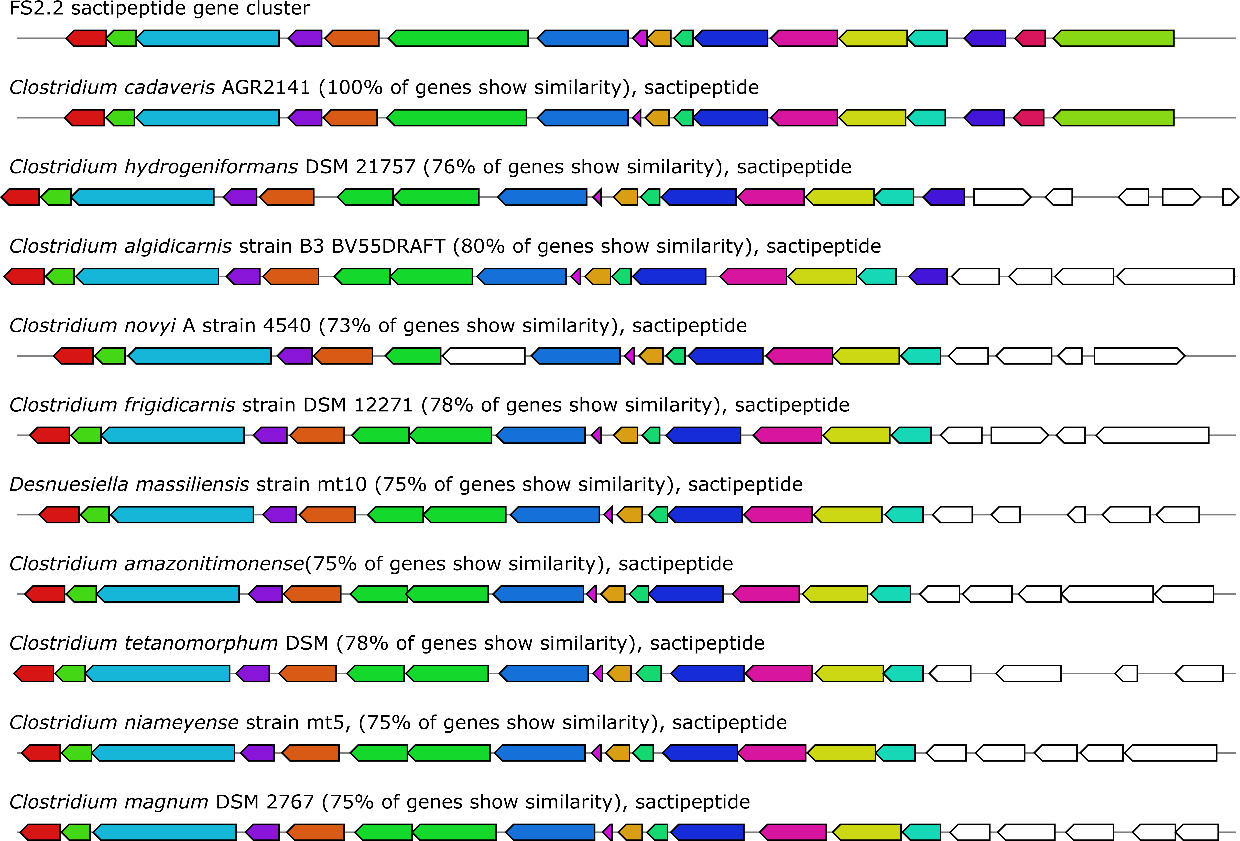
**b**


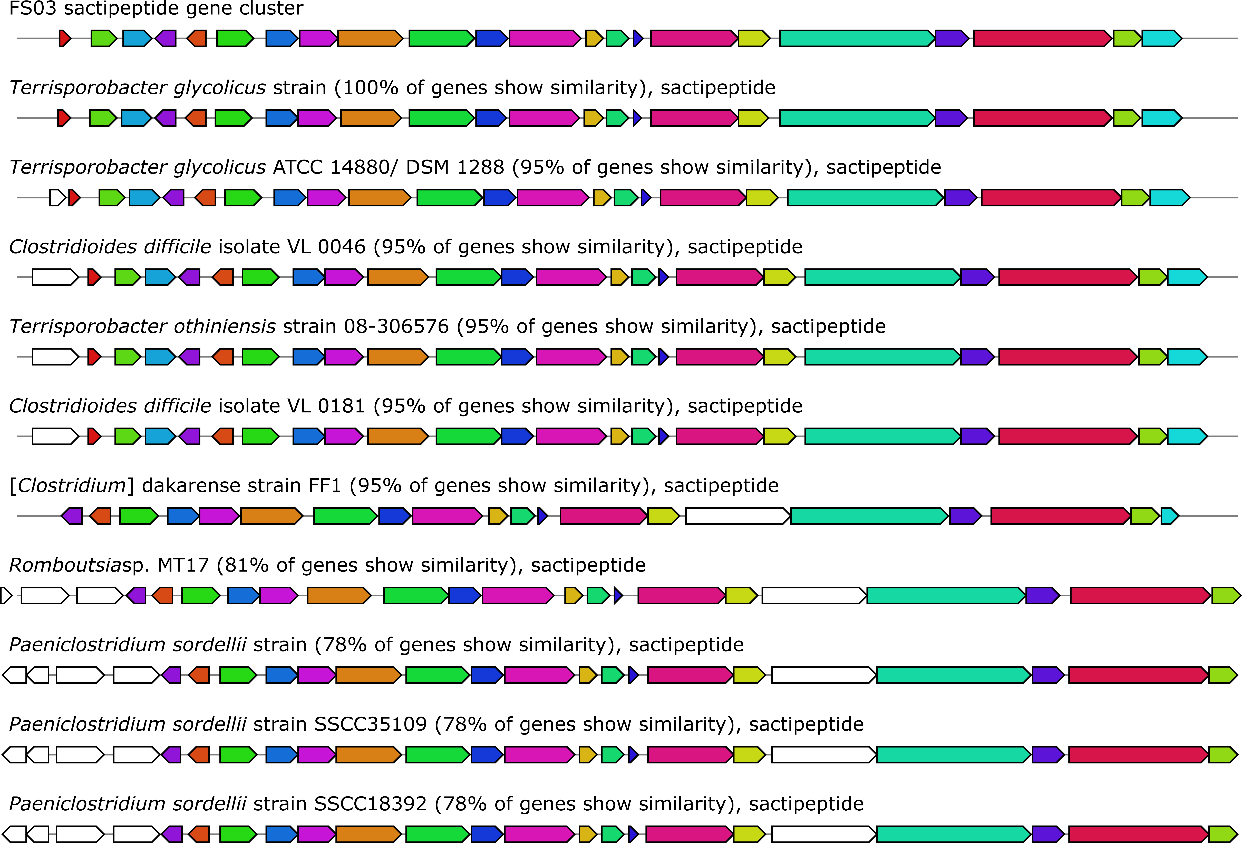
**c**

**d**


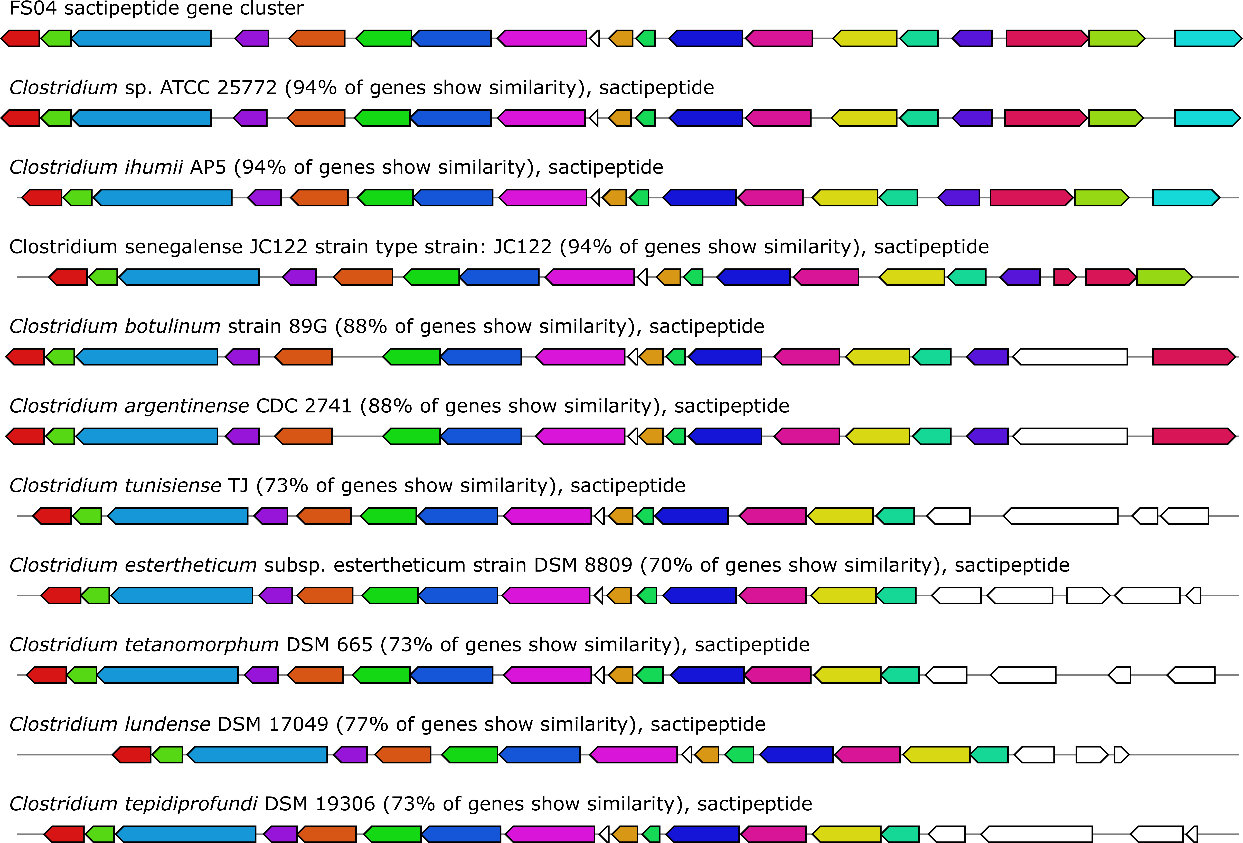

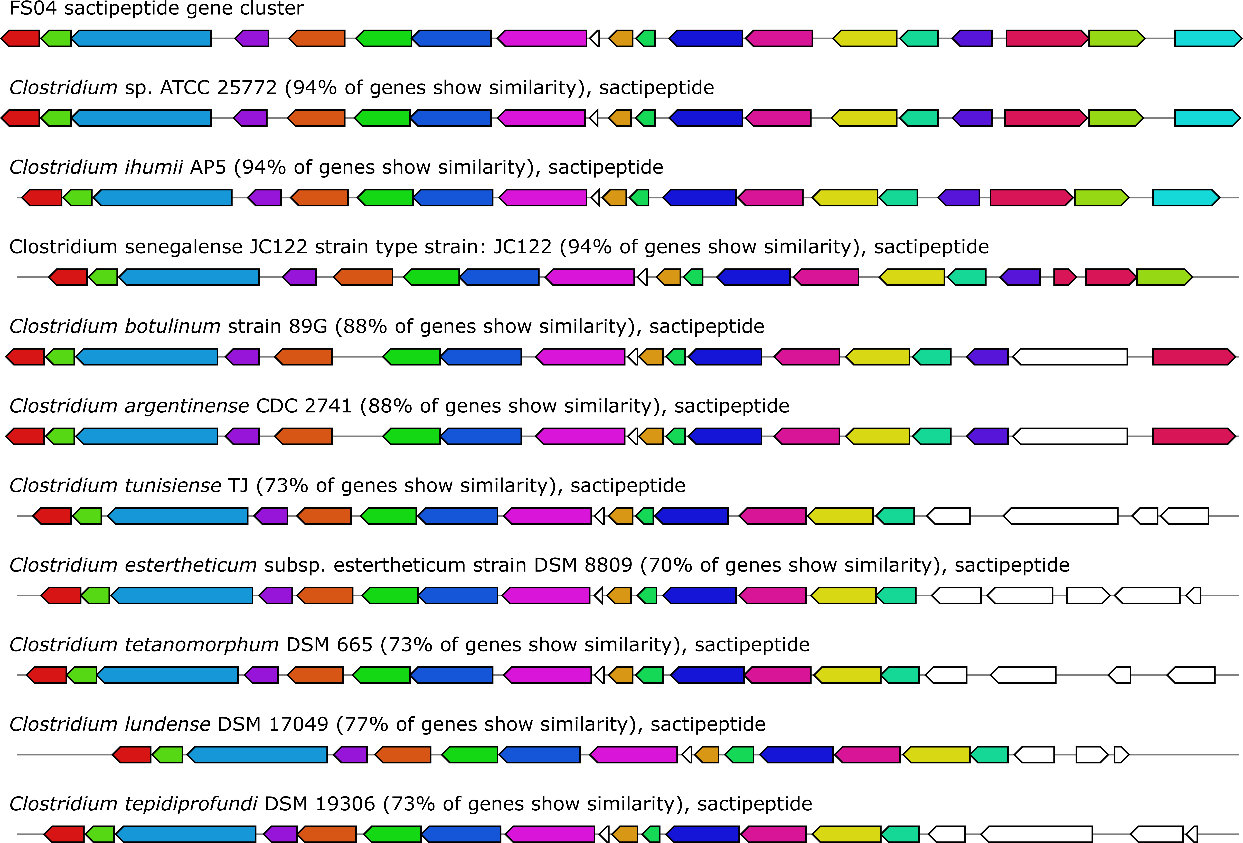


**Fig S1** Homologous gene clusters of FS01 (a) FS2.2 (b) FS03 (c) and FS04 (d) sactipeptide gene cluster. Genes with the same colour are putative homologs based on significant Blast hits. Top five matches from antiSMASH database are shown with the bacteria name and the similarity percentage.


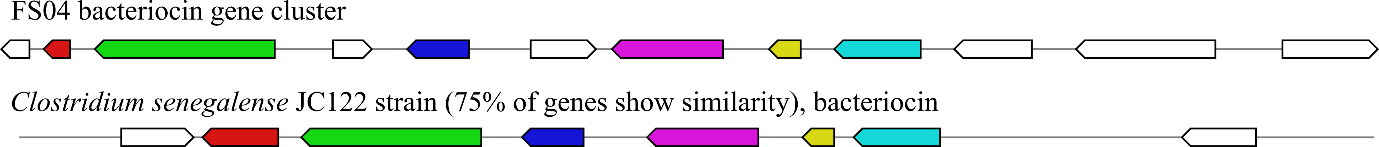


**Fig. S2** Homologous gene clusters of FS04 bacteriocin gene cluster. Genes with the same colour are putative homologs based on significant Blast hits. Best match cluster from antiSMASH database is shown with the bacteria name and the similarity percentage.
